# Reflections on using Talanoa methodology to engage with Pacific youth in Aotearoa New Zealand about their sexual and reproductive health

**DOI:** 10.1080/26410397.2024.2445934

**Published:** 2025-01-10

**Authors:** Radilaite Cammock, Tengihia Pousini, Malcolm Andrews

**Affiliations:** a Project Lead, “Let’s talk about it! Project”; Principal Investigator, School of Public Health and Interdisciplinary Studies, Faculty of Health and Environmental Studies, Auckland University of Technology, Auckland, New Zealand.; b Researcher Associate, “Let’s talk about it! Project”; Project Manager, School of Public Health and Interdisciplinary Studies, Faculty of Health and Environmental Studies, Auckland University of Technology, Auckland, New Zealand; c Researcher Associate, “Let’s talk about it! Project”; Research Officer, School of Public Health and Interdisciplinary Studies, Faculty of Health and Environmental Studies, Auckland University of Technology, Auckland, New Zealand

**Keywords:** Pacific youth sexual and reproductive health, sexual and reproductive health education, sexual taboos, Pacific sexual cultural sensitivities, *Talanoa* methods

## Abstract

Pacific understandings of sexual and reproductive health (SRH) encompass beliefs and practices reflective of Pacific values systems. These are integral to cultural understandings of safety, relationships, and intimacy. Research processes and practices that appropriately address these values and sensitivities are scarcely available in the literature, leading to limited use and understanding of culturally appropriate methods and procedures. Pacific methodologies like Talanoa are useful in ensuring that cultural perspectives unique to Pacific youth are addressed and appropriately represented in the research. This paper describes how cultural factors associated with sexual and reproductive health influenced the Talanoa research processes in a study of Pacific youth and SRH education. Key cultural considerations are discussed focussing on the positionality of the researchers, cultural sensitivities and protocols, communication strategies and the role of flexibility in privileging Pacific youth voices.

## Plain language summary

Pacific youth's sexual and reproductive health is complex. Researchers need a Pacific perspective. This paper looks at Pacific researchers working with youth in South Auckland, New Zealand. It looks at their backgrounds, experiences, and the cultural ways they work with Pacific young people. Also, it highlights how they respond to the different needs youth have. They used Talanoa, a Pacific storytelling method, for sensitive discussions. Talanoa helps in connecting with youth and understanding their cultural views. The paper shows how researchers working with Pacific youth must be respectful and supportive during Talanoa sessions. This approach builds trust and strengthens relationships. The paper provides some ideas about how researchers can work better with Pacific youth in meeting their sexual and reproductive health needs.

## Résumé

À travers le Pacifique, les conceptions de la santé sexuelle et reproductive (SSR) englobent des croyances et des pratiques qui reflètent les systèmes de valeurs du Pacifique. Elles font partie intégrante des conceptions culturelles de la sécurité, des relations et de l’intimité. Les processus et pratiques de recherche qui abordent ces valeurs et sensibilités de manière adaptée sont rarement disponibles dans les publications, ce qui conduit à une utilisation et une compréhension limitées des méthodes et procédures culturellement appropriées. Les méthodologies en usage dans le Pacifique comme Talanoa sont utiles pour veiller à ce que les perspectives culturelles propres à la jeunesse du Pacifique soient correctement abordées et représentées dans la recherche. Cet article décrit comment les facteurs culturels associés à la santé sexuelle et reproductive ont influencé les processus de recherche Talanoa dans une étude sur la jeunesse du Pacifique et l’éducation en matière de SSR. Les principales considérations culturelles sont étudiées en se concentrant sur la position des chercheurs, les sensibilités et protocoles culturels, les stratégies de communication et le rôle de la flexibilité pour privilégier les voix des jeunes du Pacifique.

## Resumen

En el Pacífico, la comprensión de la salud sexual y reproductiva (SSR) implica creencias y prácticas que reflejan los sistemas de valores del Pacífico, que son fundamentales para la comprensión cultural de la seguridad, las relaciones y la intimidad. En la literatura, escasean los procesos y las prácticas de investigación que abordan debidamente estos valores y sensibilidades, por lo que el uso y la comprensión de métodos y procedimientos culturalmente adecuados son limitados. Las metodologías del Pacífico, como Talanoa, son útiles para procurar que las perspectivas culturales únicas de jóvenes del Pacífico sean abordadas y representadas debidamente en las investigaciones. Este artículo describe cómo los factores culturales asociados con la salud sexual y reproductiva influyeron en los procesos de investigación de Talanoa en un estudio sobre jóvenes del Pacífico y educación en SSR. Se examinan consideraciones culturales clave, con un enfoque en la posicionalidad de los investigadores, las sensibilidades y los protocolos culturales, las estrategias de comunicación y el papel de la flexibilidad en privilegiar las voces de jóvenes del Pacífico.

## Introduction

Within the Pacific region,* discussing sexual health with young people involves talking about themes like sex, intimacy, gender identity, reproduction and fertility.^1–5^ These topics are confronting and challenging where cultural sensitivities and taboos exist.^6^ Furthermore, the internet has increased the accessibility and availability of information, becoming one of the most influential sources of sexual health information for young Pacific people.^7^ In these contexts, young people are challenged by social pressures that set expectations on individuals’ sense of self and identity.^8^ Within the Pacific, supporting young people to manage and discuss their sexual or reproductive needs can be difficult.^9^ Research by Cammock *et al.*^1^ found that getting young people to raise concerns with their health provider is often problematic. These challenges extend to barriers that involve accessing sexual and reproductive health services like STI protection and contraception.^1^

Within the home, raising these issues with their families can pose challenges for Pacific youth due to a fear of being “told off” or stigma.^1,10^ These challenges are further enhanced when factoring in cultural taboos and sensitivities around relationships, gender, sexuality and reproduction.^9,11,12^ Research amongst young Itaukei (Indigenous Fijian) women found that raising issues or questions with Fijian parents was difficult for them, due to the reverent relationship between fathers and daughters and mothers and sons, which can lead to an avoidance of sexual and reproductive health discussions in the home.^1,13^ Within research these complexities impact the lens through which investigations are explored, analysed, and understood. These factors require research approaches to be multidimensional and holistic in nature, accounting for social and cultural norms that impact participant involvement. In this paper, we outline the “Let’s talk about it!” project and our reflections on the use of Talanoa as a methodology for engaging with Pacific youth. This article discusses the cultural factors associated with carrying out sexual and reproductive health research in general and possible implications for future research with Pacific youth.

### The “Let’s talk about it!” project

In 2020, we started a research project looking into the sexual and reproductive health education of Pacific youth based in Aotearoa New Zealand and used a talanoa, a form of dialogue commonly practiced in the Pacific, to discuss Pacific youth experiences and perspectives.^9^ The research project recruited eight Pacific youth attending a high school in South Auckland where a high proportion of Pacific peoples in Aotearoa New Zealand live. The study included four Pacific females and four Pacific males aged between 16 and18 years.^†^ We carried out six Talanoa sessions over a period of three months. Talanoa sessions were one and a half hours long, and sessions were audio-recorded for analysis purposes. To ensure ease of access for students, the research took place in classrooms at the high school that students attended. The sessions were carried out in the evening and students were provided with food during the sessions. Students were also given information and consent forms, outlining confidentiality considerations and details of support services available to them if needed (Auckland University of Technology Ethics Committee Ref 20/263, Granted 12/10/2020). Confidentiality, privacy and any issues around comfort were discussed in our information sessions prior to Talanoa sessions taking place. For more information on the findings of the study please see Cammock *et al.*^9^ Below we detail the Talanoa process.

## Talanoa methodology

It is important to note that the Pacific region is home to many languages and cultures. For Pacific indigenous people, cultural traditions and protocols are embedded in everyday living. Talanoa is an indigenous form of storytelling to many ethnic groups in the Pacific. It has also been widely used as a qualitative research method and approach within Pacific research.^14–16^ Decisions on the design and approaches within Talanoa are influenced by ethnic specificity and the associated Pacific cultural lens which dictate methods of inquiry and analyses. Talanoa approaches contribute to indigenous research methodological movements (IRM) that advocate for a shift in the research paradigm amongst indigenous people.^17^ IRM encourages researchers to move their focus away from western theories to ethnocentric perspectives and knowledges. Smith^17^ advocates for a need for a more significant understanding of conventions and assumptions that form the basis of indigenous values and motivations. Indigenous values and practices uphold the rights of Pacific people by ensuring that Pacific identity is reinforced through research processes and approaches. This involves the incorporation of values such as *vakarokoroko* or respect and *veitokoni* or reciprocity, which enhances a researcher’s ability to engage with Pacific participants in more meaningful ways. This is critical to ensure that conceptualisations of the impact of research findings and outputs on Pacific peoples’ well-being are considered. If carried out appropriately these approaches could lead to the decolonising of research agendas among Pacific people.^17^

Within a Eurocentric positioning, Talanoa has roots in phenomenology whereby phenomena are understood through participants’ personal knowledge, experience and subjectivity.^18,19^ Within a Pacific-centric paradigm, Talanoa is rooted in indigenous methods of enquiry that foster values of *veiwekani* (relationship) and personal engagement, placing ontological considerations of Pacific indigenous cultures central to investigations.

Conducting Talanoa within Pacific spaces requires a connection and understanding of the emotional and cultural complexity that comes with Pacific enquiry.^20^ Tecun *et al.* explain:

*“When incorporating Talanoa in research settings, one must expect emotions to be connected to this form of knowledge and understanding. Talanoa is an embodied expression of the Fijian Vanua, a concept that includes love, empathy and respect. As an indigenous method of learning and enquiry, it creates closeness rather than distance within an assumed objectivity that is commonplace in dominant Western research practices.”* (p.158)^20^

Talanoa removes the gap between the researcher and participant and encourages empathetic research approaches.^14^ Although similar to narratives, Talanoa goes beyond just storytelling to providing participants challenging courses of communication where participants and researchers probe, verify and legitimise each other’s stories.^19^ This is especially true for group discussions where participants have the opportunity to challenge and confirm phenomena.^19^ In this way Talanoa is not an extractive process of information gathering, rather a process whereby engagement with Pacific peoples is necessary and where reciprocal exchange is critical.^20^

## Talanoa considerations within the “Let’s talk about it!” project

Within the “Let’s talk about it!” project, key cultural values pertaining to sexual and reproductive health influenced how Talanoa processes and methods were carried out. Given the Pacific taboos around SRH and the holistic positioning of reproduction within the family unit,^21^ it was important that discussions moved beyond sex or sexual intimacy, to topics of fertility, teenage pregnancy and STIs. The socio-cultural context also became critical to explore in light of the social pressures that youth experience, and the impact of social media on self-esteem and identity.^7,10^ These considerations were integral in the planning and scope of how research questions and methods were positioned. Specifically, Talanoa methodological considerations included the (1) positionality of the research team, (2) sensitivity to cultural practices and protocols, (3) communications strategies, and (4) privileging the youth voice through flexibility.

### Positionality of researchers

Within Pacific research, situating oneself in relation to the research topic, aims and context is critical in understanding the lens through which the research is being conducted. Within the Pacific, researcher positionality and reflexivity traces connections back to tradition and oceanic relationships between island nations, clans and people.^22^ Whether through kin or contemporary geographic proximation, Pacific peoples share a history that spans Indigenous and postcolonial histories.^22,23^ Within these realities come avenues for shared understanding, appreciation and commitment to a shared goal and vision.^24^

To ensure empathetic Talanoa was possible, being mindful of one’s connection to the research context and therefore participants was critical. The authors of this article facilitated Talanoa interactions and discussions with Pacific youth. Below we discuss each author's connection to the Pacific and the research context.

Radilaite Cammock: I am from the village of Muanaira Vutia, in the Rewan province of Fiji. I attended high school in Aotearoa. Sexuality education was available at my high school, but my parents were not comfortable with teachers providing this information. Although this led to limited exposure and therefore awareness of sexual or reproductive health information during my adolescent years, I became conscious of varying views on SRH and more particularly the generational differences in how culture and SRH taboos influenced young people’s attitudes and exposure to SRH.

Tengihia Pousini: I was born in Aotearoa New Zealand with lineage tracing back to the villages of Pea, Ma’ufanga and Matahau on the island of Tongatapu, Tonga. Growing up in a traditional Tongan household, I shared an understanding of taboos surrounding SRH education that align with the ethos of *Faka’apa’apa* or respect. Upholding *Faka’apa’apa* led to some challenges in accessing comprehensive SRH information during high school. Although I participated in one sex education class, I felt hesitant to ask questions or engage in discussion with educators. Instead, I preferred getting advice from female peers who had prior experience in accessing SRH services. This dynamic resonated with other Pacific youth in the study, who shared similar patterns of health-seeking behaviours.

Malcolm Andrews: I am from the villages of Wainakola and Vagadaci in the Lomaiviti province, and Makolei, Kasavu and Savusavu in Fiji. I was born and raised in Fiji before migrating to New Zealand to complete my senior years of high school. I only attended a single SRH education session in Fiji and the learnings were not discussed ever again in school nor at home. I felt awkward and lacked the skills needed to discuss SRH openly while in high school in Aotearoa NZ. These challenges included conversing about a sensitive topic inside a classroom with cousins/family/village/church members of the opposite sex, while also being aware and grounded in key Fijian values such as *Veirokorokovi* (respecting one another) and *Tabu* (taboo). This experience was also evident with some Pacific youth in the study.

#### Navigating insider/outsider status

An awareness of the Pacific facilitators’ insider status facilitated a process whereby shared understandings with youth of their lived realities and experiences were possible.^25^ All three researchers had strong connections to their cultural heritage and were able to understand varying perspectives that youth had that were specific to the Aotearoa New Zealand context. Given this, being part of a research team and therefore “outside” or “apart” from the participants was also an identity that researchers navigated. Navigating these positions points to a nuanced framing within research, called straddling, where researchers have similar attitudes and experiences to participants while also pursuing new knowledge and understanding. Our experience is what Masters^25^ refers to as a sense of sameness and difference in researcher experience.^25,26^

Navigating the insider/outsider dichotomy respectfully requires engagement and meaningful relationships with participants. Within the “Let’s talk about it!” project, forming relationships was possible through an understanding of the Pacific values and cultural practices that we shared as Pacific youth growing and learning in Aotearoa NZ. These experiences ensured a mutual respect or *vakarokoroko* for the lived realities of youth and the questions and criticisms that were being raised in Talanoa.^27^ These connections ensured that *veiwekani* or relationship building occurred prior to, during and after Talanoa sessions occurred. These included food and gifts of appreciation after Talanoa sessions. Understanding the relational responsibility^28^ of researchers and facilitators and the role participants play in the research leads to transparency and accountability.^29,30^

### Sensitivity to cultural practices and protocols

The way in which researchers acknowledge cultural protocols and obligations of a particular group is critical in Pacific research.^14,15,31–33^ Latai-Niusulu^34^ found that conducting research in Samoan as a Samoan researcher meant that the use of *va-tapuia* or behaviour norms and interactions between people and places established connection and engagement with participants in the *aiga* or family, *fono* or village councils. In her research, researchers ran the risk of encountering problems if cultural protocols and obligations were ignored. She warned that relationships worsened where cultural practices like *utu*, *totongi* (reciprocation of unsuitable or wrong responses) were performed by participants where participation contributions were diminished, limiting their level of engagement, and the reliability and quality of findings.^34^

#### Cultural values associated with SRH research

There are various cultural practices and belief systems that researchers need to be cognisant of when working with Pacific youth and discussing sexual health topics. It is important to note that within our study, Talanoa groups were made up of five Tongan, two Fijian and one Samoan student. The multiethnic nature of the group required an understanding of values across all three ethnicities. These include *tabu* in the Fijian language, *tapu, noa* and *tauhi va* in the Tongan language and *teu le va* in the Samoan language. Critical to these discussions were the gender implications within Talanoa groups and the Pacific spaces, relationships and family constructs traversed.

## Tabu, Tapu, Noa

A key value within SRH is *tabu* in the Fijian language or *tapu* in the Tongan and Samoan languages. These values are defined by “that which is sacred, restricted or set apart”.^20,21^ To gain a clearer understanding of how *tabu* or *tapu* apply in SRH, an understanding of specific cultural practices is required. Within Fijian contexts, topics of the sexual nature are considered *tabu* due to the role that sexual intimacy has in reproduction and the divine role women have in bringing life into the world, a role that is analogous to a higher ecclesiastical being that brings life to the earth. This association commands a level of sacredness, and respect when discussing SRH in Pacific spaces.^1^ Within Tongan traditions, what is *tapu* is similarly considered divine and noble and denotes *mana* or power and authority.^20^ In the context of SRH, *mana* can refer to the strength of one’s identity and sense of self.^21^ Within Tongan traditions *noa* is often seen to accompany *tapu*, as a way to restore balance or make something that is *tapu*, safe,^20,35^ Young *et al.* discuss *tapu* and noa within the context of sexual health as darkness and light. *Tapu* in this sense refers to elements of sexual health that are unknown, less understood and therefore difficult and challenging to navigate. Reaching a place of *noa* symbolises attaining a level of safety that involves having the space to learn and grow in one’s understanding of sexual health desires.^35^ Tecun *et al.* explain:

*“Noa, resulting from having neutralised the tapu between people, gender, rank, circumstance, and place, is where beautiful artistic expression or knowledge can be openly shared.”*^20^

Within the “Let's talk about it!” project, reaching a level of *noa* was a critical point during engagement and relationship building. During Talanoa sessions *noa* was achieved when youth felt comfortable in their relationships with other members of the groups as well as with the researchers. This level of comfort allowed youth to share their perspectives freely.

### *Tauhi va, teu le va* – nurturing relationships

The nurturing of relationships or *tauhi va* in Tongan,^20^ *teu le va* in Samoan,^36^ and *veiwekani* or *veimaliwai* in Fijian led to the attainment of *noa* in our study, by ensuring that the *tapu* or *tabu* associated with SRH was mediated. To ensure *tauhi va*, the concepts of time and space or *tā va* are critical to note. *Tā va* refers to the time and space and the intersections between people. *Tauhi va* further denotes the nurturing of the space between individuals. ^20^ *Anae*^36^ approaches *teu le va* in the Samoan context as a process of valuing and nurturing the reciprocal relational spaces that permeate Pacific research and the communities and peoples that are involved.

To nurture the relational space, one requires an understanding of the individuals that the space connects and the *mana* they bring to the research. Understanding the context in which participants live and operate enables researchers to connect for subsequent interactions, relationship building and nurturing. For the “Let’s talk about it!” project, our project lead was from the community where the project was based and had spent about a year and a half working with young people in the community prior to the study commencing. The researchers were also familiar with the community, which helped build connection with participants. This connection extended after Talanoa sessions were completed and involved youth having input during the data analysis phases of the project.

In the study, group participants were purposively invited if they were friends with other members of the group, to ensure that a relationship was established and that participants were familiar with each other. This helped establish *noa* within the Talanoa process where youth felt safe and comfortable to share their views. Tecun *et al.* explain:

*“If relations are already established and well maintained or a setting is a noa space that is safe and calibrated, then noa may take less time to reach and less extensive levels of protocol. However, new relationships or settings may require additional formalities such as type of dress, gifting, preparing food, eating food, or another such activity that can render the sacredness of each individual to calibrate in a good relational way.”*^20^

Resolving power imbalances when working with Pacific youth is an essential component of *tauhi va* and reaching a place of *noa* in Talanoa. For our researchers, a shared identity and understanding of growing up and being exposed to new social norms in Aotearoa, allowed connections to form, and greater appreciation for youth and their experiences.^9,25^

#### Gendered configuration of Talanoa sessions

Within Pacific research, gendered *tabu* or *tapu* associated with the roles of male and female individuals in Pacific families and communities heavily dictate research processes. Within a Pacific worldview, traditional roles of men and women were aligned with their contributions to the collective, hence the contribution of women was aligned with their ability to reproduce and nurture, while for men it was to protect and provide.^37^ Respect for these roles often meant that within family units, young girls were cherished and treated with reverence in interactions with their male siblings. The *tapu* nature of reproduction and the role of women in bearing children further amplifies the sacredness of girls and women and the importance of nurturing relationships with members of the opposite sex. These practices remain evident in the way male and female youth interact within family and community life.^13^ Given the key role gender has within the context of sexual and reproductive health discourse, having gendered discussions was considered the appropriate format for Talanoa with Pacific youth in our study.

In Young *et al.*’s consideration of *tapu* within SRH research processes, gendered *tapu* was highlighted as an important consideration when consulting with advisory groups.^35^ These factors ensured that participants felt comfortable and acknowledged during the Talanoa discussion. With the “Let’s talk about it!” project, male researchers facilitated male Talanoa sessions while female researchers facilitated female Talanoa sessions. Having researchers and facilitators of the same gender ensured that reciprocal exchange was carried out in a manner that acknowledged gender roles and considerations within Pacific spaces. Given this, opportunities for both genders to come together were also provided. These occurred once gendered Talanoa sessions had been carried out. These considerations were also determined by the comfort levels of male and female participants. Providing avenues for both gendered Talanoa and mixed Talanoa ensured that as a research team, we were able to fully explore and capture the dynamics between participants in Talanoa sessions.

### Communication strategies

Face-to-face communication is vital in ensuring that authentic reciprocal exchange occurs.^19,38^ In our study, face-to-face Talanoa provided a culturally appropriate mode in which research could be carried out, as Pacific knowledge, culture and traditions are more often perpetuated through oral communication rather than written.^39^ In our study, face-to-face communication and interaction ensured that as researchers we were able to pick up on non-verbal cues that depicted comfort levels for youth and whether youth agreed or not with their peers.^40^

#### Navigating language competencies associated with sexual and reproductive health

Language competencies were crucial in Talanoa with Pacific youth. Otsuka explains that indigenous Fijians often did not verbalise simple indications like yes, or no or whether they agree or disagree, a response he explained was due to the researcher not speaking the same language.^32^ Therefore, in his study, being familiar with the indigenous language was useful in communicating with participants. Otsuka also found the way in which Fijian people spoke to be initially ambiguous and indirect. He explains that language and communication differences may contribute to this and advises researchers to be aware of different ways in which participants express particular experiences and emotions.^32^ In Latai-Niusulu’s study on community-based investigations in Samoa, the researchers discuss the respect that young people showed towards elders, known as *faaaloalo i e matutua*, whereby youth were non-verbal and silent in the presence of elders.^34^ Within our study, group Talanoa sessions were considered more appropriate compared to one-on-one Talanoa sessions, to acknowledge the respect and potential intimidation that young people could feel when speaking to facilitators. Furthermore, group configurations provided youth with more opportunities to respond to each other and raise questions when sharing perspectives.

The taboo nature of SRH topics also meant that certain SRH terms needed to be refined and considered when creating question schedules for Talanoa sessions. In Young *et al.*'s ^35^ study of Pacific university students, open-ended questions were preferred over set response questions to allow for various responses to SRH questions.^12^ The Pacific researchers avoided using words like “pornography”, “sexual orientation”, “rape” or “abuse” and instead asked questions pertaining to “sexual wellbeing”. Within our study, the first Talanoa we had with youth involved a brainstorming exercise to determine the language and breadth of understanding youth had of their sexual and reproductive health. Through this exercise, we were able to identify the terms and concepts youth were aware of, and the appropriate terminology to use in Talanoa sessions. The subsequent Talanoa sessions with youth were framed around the key ideas, themes and topics raised in the brainstorming activity. Youth discussed harassment, cultural *tabu*, teenage pregnancy and STI in the brainstorming activities, presenting us with sexual health topics for further exploration in subsequent Talanoa sessions.

### Informal Talanoa versus formal Talanoa

Vaioleti discusses an informality to Talanoa which enables open speech and conversation.^41^ Tecun *et al.* argue that “informality” may play a role in establishing relationships and addressing tensions between age, gender, rank, and religious affiliation.^20^ They explain informal Talanoa to be critical in providing the space or “*va”* for more “formal” conversations to transpire. In our study, informal Talanoa was undertaken prior to formal Talanoa to establish the *va.* This made youth feel more comfortable to share their perspectives and experiences. Thus an acknowledgement of Pacific epistemology and realities leads to an acknowledgement of how integral “informal” processes are leading up to “formal” Talanoa. Building relationship and engagement with youth was critical to get youth to a place of “openness” where Talanoa was free-flowing. When building relationships with youth any tensions with rank, ethnicity, age and gender were explored through “informal” Talanoa prior to “formal” data collection methods. This enabled youth participants to reach an understanding of their roles in the Talanoa session and consider the shared vision and goals of the Talanoa.

### Youth voice and the need for flexibility

The practicalities of carrying out Talanoa within Pacific research involve flexibility and versatility. Given the multi-ethnic configuration, ages and backgrounds of participants, and shifting socio-cultural makeup of Pacific identities in Aotearoa, Talanoa processes and methods needed to be adaptive to changing circumstances and experiences. Tecan and colleagues explain that

*“Talanoa requires movement, at times spontaneity, and can take place nearly anywhere. Talanoa is not static and should be carried out with an understanding that local knowledge systems are perpetually negotiated as living cultures that are continually evolving.”*^20^

Within our youth Talanoa, there were moments where facilitators needed to be flexible to move between serious topics to those considered less serious in nature. For example, discussions that focused on friendships involved humour, whereas topics of sexual harassment were treated with more gravitas. Youth in these sessions were guided by the tone and approach of the facilitators, illustrating the skill level needed in talanoa to navigate discussions.^9,16^

Youth in our study varied in age and background. Some had parents living in their home Island countries while others were brought up by siblings and extended family members in Aotearoa New Zealand. Youth discussed parents who had experienced teenage pregnancies or sexual harassment while others were more aware of religious expectations and traditional roles of men and women in the home. These experiences highlight a reality for Pacific youth in Aotearoa, where grappling with cultural expectations alongside trying to meet mainstream social pressures is commonplace.^10,37^ As researchers, this meant that we needed to be mindful of avoiding leading Talanoa discussions away from what might be relevant for youth. We focused on ensuring that youth drove the Talanoa and that any probes into cultural influences were responsive to narratives being shared by youth. Recognising these nuances and tensions is critical in building the skills of a Pacific researcher in the SRH space.

This flexibility extends to cultural protocols like *lotu* or prayer that may be confronting for youth who are not religious. In the current study, although Talanoa sessions opened and closed with a prayer, researchers were careful to ensure youth were comfortable with sharing these practices in the Talanoa space. Fa’avae *et al.* explain that traditional Pacific research protocols like prayer may not necessarily align to the norms and practices of research communities, particularly when cultural norms impact youth and their constructions of social normalities outside of traditional structures e.g. village, or churches.^42^ In quoting Hau’ofa^43^; Marsters^25^ states (p.83):

*“We’ve often put our traditions in cages, and so we try to do what we think our elders, the people in the past, did. And we trap our traditions there. We freeze them. Whereas people in the past really lived very much like people in the present. There were always cultures mixing. Things were fluid, they were not frozen. But we froze them.”*

Such careful appreciation for the context in which Pacific youth grow up and their mixed cultural identities leads to a more in-depth understanding of research needs when conducting culturally sensitive SRH research. This creates authentic Talanoa that is framed around the critical ontological values and practice systems of Pacific youth.

## Discussion and conclusions

Pacific youth are a marginalised group in Aotearoa with poor SRH outcomes.^44,45^ Carrying out research amongst Pacific youth requires sustained relationship building and meaningful partnerships. Globally, research shows that providing vulnerable communities the opportunity to engage in research in this way removes the barriers to research participation and provides more opportunities for ethical research practices.^46^ Reducing “risk” amongst vulnerable groups in research also requires researchers to value participation from youth appropriately.^47^ In their research on ethical practices in sexual health, Fliker and Guta discuss the provision of honoraria to survey youth participants in the form of condoms or movie tickets. Demonstrating the value added by participants.^48^ For Pacific youth, we argue that this is reflected in reciprocal engagement and acknowledgement of cultural protocols like *teu le va,* where demonstrating participants’ value through research Talanoa and the offering of food and gifts reinforces and supports youth empowerment and identity. Furthermore, the need for constant reflexivity and researcher positionality allows researchers to be adaptive and responsive to youth voice and needs throughout the research process.^49^

### Unpacking cultural taboo

Current understandings of Talanoa amongst youth are drawn from studies that are limited in their exploration of cultural practices associated with SRH research. Although cultural taboos are referenced in the literature as a significant barrier to accessing and discussing SRH amongst Pacific youth,^6^ little is known about how to incorporate cultural understandings into methodological designs. Researchers working in the sexual and reproductive health sector are reliant on Eurocentric methodologies and methods to carry out research amongst Pacific youth. This article provides key cultural considerations that researchers can incorporate into their research designs to help engage, build relationships and ensure cultural safety for both the researcher and youth participant.

For example, we provide a deeper understanding of *teu le va* and the role it plays in ensuring recruitment processes with Pacific youth go beyond “transactional” arrangements to arrangements that are more relationship-based.^16^ Table 1 provides a summary of the Talanoa approach used in the study and the key Pacific research values and considerations when engaging with Pacific youth. The table highlights key research implications to assist future researchers working with Pacific youth within the sexual and reproductive health context.

**Table 1.** Talanoa considerations and implications for research

Talanoa considerations	Pacific framing (values)	Implications for research process
Positionality of research team	• Historical and genealogical connection to the Pacific • Reciprocal engagement with participants and respect for participant • Identification of “Insider” and/or “outsider” experiences	• Identifying points of connection and relational positionality • Building relationships with youth Participants • Removing power imbalance • Providing food and gifts to acknowledge participation of participants
Sensitivity to cultural practices and protocols	• *Tabu, Tapu, Noa* – understanding cultural taboo • *Tahi Va, Teu le va* – nurturing relationships • Implication of Gender roles and responsibilities on group Talanoa • Valuing youth and their “*Mana”* or identity	• Treating taboo topics with respect • Establishing a “safe” space for youth to Talanoa • Ensuring participants are comfortable to share experiences • Recruiting friends for group Talanoa to ensure connection and comfort of participants • Opportunity for both gendered and mixed Talanoa sessions
Communications strategies	• Pacific language competency • Awareness of verbal and non-verbal cues (silence) • Identifying need for Informal and formal Talanoa	• Awareness of non-verbal cues and body language. • Understanding that silence has meaning • Pacific framing of SRH terms e.g. pornography, rape, abuse • Face to face communication • Preparation for formal Talanoa using informal Talanoa
Privileging youth voice and flexibility	• Inclusivity of diverse youth experiences • Influence of generational differences and the role of traditional values	• Consideration of demographic changes and youthful populations • Providing youth with opportunity to have input into research process, analyses and findings • Understanding of youth realities and influence of new social norms

### Rights based approaches and cultural safety

The research also highlights the criticality of incorporating rights-based approaches in sexual and reproductive health research amongst Indigenous or vulnerable youth. The cultural practices highlighted in this article, although grounded in Pacific Indigenous knowledge, have significance to broader discussions of rights-based approaches in SRH research, especially amongst vulnerable marginalised groups. For example, *vakarokoroko* or respect for the “space” or *Va* in research relationships echoes the notion of respect that adolescents expect from their peers and guardians.^50^ Findings from Berglas *et al.*’s^51^ study on Hispanic and African American youth, showed that exercising rights in relationships was reflective of individuals’ self-respect, while acknowledging others’ rights in a relationship was a sign of mutual respect.^51^

The focus on rights-based approaches leads to cultural safety in SRH research. Cultural safety has been described as a focus on the delivery of care and an acknowledgement of power relationships and patients’ rights.^52^ This definition seeks to question differences in the researcher-participant relationship and any power imbalance that might exist. Thus, operating in a culturally safe way recognises the rights that participants have during the research and, in turn, the responsibilities of researchers to address biases, assumptions and any prejudices they might have in the relationship.^53^ Within Talanoa these considerations are at the heart of key cultural practices that focus on ensuring participants feel safe within the *Va* space when Talanoa is taking place. It also requires the participant and their experiences to be central to research processes.^54^

In conclusion, our experiences highlight the complexity involved with understanding sexual taboos and cultural protocols. We argue for the importance of building relationships with youth and ensuring that Talanoa discussions are carried out with the appropriate flexibility and fluidity that allows varied experiences, comfort levels and sensitivities to be included. An appreciation for the youth voice and their centrality to the research provides a focal point for Talanoa methodological decisions.

## Footnotes

* Constitutes small island nations in the Pacific region which includes countries in Melanesia, Micronesia and Polynesia. Melanesian countries are those situated in the southwestern part of the Pacific Ocean and include Vanuatu, Solomon Islands, Papua New Guinea and Fiji. Countries in Micronesia are situated in the northwestern regions of the Pacific Ocean and constitute countries like Kiribati and Nauru. Polynesian nations constitute those that occupy the southeastern part of the Pacific Ocean and include Samoa, Tonga, Cook Islands and Aotearoa New Zealand.

† Although Pacific youth who identify with a third gender were considered, we did not have youth from this group volunteer to be part of the study.
